# Germs Not Pathological

**Published:** 1894-06-23

**Authors:** 


### The Hospital

AN INSTITUTIONAL JOURNAL OF

The Medical Sciences and Hospital Administration,

WITH

AN EXTRA NURSING SUPPLEMENT.

VOL. XVI.—No. 404.] [JUNE 23, 1894.

### GERMS NOT PATHOLOGICAL.

If it be true of all men that "they cannot live by bread alone," so it is true of all doctors that they cannot exist and be happy on an exclusive diet of physic. Medical science makes large demands upon the intellect; and medical practice insists upon an excessive share of the physical energy. But though a man may and must satisfy these claims to the full, they cannot, in their turn, give entire contentment to him. The doctor is a man, a citizen of his state, and a brother to his fellow men, as well as a student and a practitioner of the healing science and art.

The germs of political life and activity are stirring in the modern doctor. It has been his metier in the past to repudiate politics; and he has suffered the usual consequences; politics have repudiated him. In the modern political world nobody is of so little consequence as the doctor. The proofs of this are self-evident, and present themselves in battalions to the least reflecting medical mind. There may have been advantages in shutting out politics from the field of medical activity in the past, and it is certain there was freedom from many worries and excitements. Even now one can quite understand why medical men of overpoweringly scientific bias may firmly resolve to leave politics alone. Science is exacting; politics are exacting. The difficulty of serving two masters and serving them both well, is proverbial. The true lover of science could not be "happy with either," even if "t'other dear charmer were away." Science is his first and last love, and to her alone he feels he can be faithful unto death.

But what a generous man or a generous profession hesitates to do for itself it will sometimes resolve to do for other people. This seems to us to be the parting of the ways at which the medical profession has now arrived. Is medicine in the future to be, as she has been in the past, of the cloister? Or is she to identify herself as an active and resolute force with all the struggles and forward movements of a progressive civilisation? There can hardly be more than one answer to this question. Just as one of the most prominent statesmen of the time has torn himself from his books and thrown aside the pen of the writer for the actual political struggles of the hour, so must medicine as a profession cease to be merely scientific and merely domestic, and apply herself to the solutions of the manifold and great social problems which a national and universal, as opposed to a merely individual, physiology alone can solve.

Physiology is a science of details. The physiologist must be a man of details. But we submit that man himself, in the whole scheme of things, is a detail; and when he happens to be an Englishman he is, from the point of view of the present argument, a very important detail. Moreover, we hold that physiology may secure such successes by judicious dealing with men in the mass as will provide her with much-needed consolations for her failures in dealing with men in the individual and in detail. We set ourselves upon the health of the individual, upon the cure of the class. Very often the individual, as the diabetic, or the class, as the consumptive, declines utterly to be cured. Then our science is frustrated, and we ourselves are grievously discouraged. But why should not the health of the whole nation and empire, the food, the sleep, the work, and the amusements, the politics and the religion, the breeding and feeding and training of a race of men of constantly-improving physical and mental capacity—why should not these be our care as physiologists? They would have been our care long ago if we had had the intellectual capacity to see our science in the large as well as in the small. The whole world does not consist of one normal man, nor of a few sick men. What the physiologist finds in one normal man he may find in the whole race; the evil which the physician perceives in the few sick he will see also, at least potentially, in all the well. A too unintelligent particularism has been the ruin of the doctor's intelligence, and has paralysed all his influence in the state. To our thinking the man of all others who has the right knowledge for guiding the masses of the people safely and wisely at the present crisis is the philosophical physiologist. But, alas! for the multitudes who need his guidance, the physiologist is the very last person in the world who either asks or could obtain a hearing.

There are signs that doctors are stirring politically. It is true the movements are in spurts; they are feeble also, and lack coherence. Their objects, moreover, are not as yet very great, but they are at least legitimate. Medical men are combining for the safeguarding of their own interests. Unfortunately, as too often happens in similar circumstances, the leaders are not in every case everything we could desire. But in that doctors are learning to act together, in that they are so far laying aside their inordinate, paralysing, and really ridiculous jealousies of each other, they are moving in the right direction. It is necessary, in the interests of those who carry on the work of this vast empire, that physiology and medicine shall be widely influential. They cannot be widely influential until medicine has learnt to speak with one voice and to act with one will. If medical men begin to combine for small objects they will go on to combine for greater. If they learn to work together for their own interests their next step will be to combine their knowledge for effective public action, and to lift up the whole people by their united physiological energy to a higher level of health, efficiency, and happiness.

It is said that among the older and generally acknowledged leaders of the profession there is a marked want of sympathy with the new political activity of the profession. This is not easily accounted for, except on the assumption of want of vitality and intellectual freshness among the older men. But if that be admitted, it is a fatal admission. No man can afford to be intellectually stale in these times. The statesman, the physician, and the man of science, whatever their ages, must be fresh as the youngest in the keenness of their intellectual appetites for new discoveries, and in their readiness to readjust their mental organisations to new developments. The price of pre-eminence is eternal freshness. If our venerable leaders look on our political activity with stony indifference or even with hesitating reserve, they will be left behind like the sphinx in the desert, whilst the resistless army of a newly-vitalised medicine will march to newer and wider victories beneath its motto of "Forward evermore."

The oldest political organisation in the profession is the British Medical Association. That Association does not appear to make quite all the efforts it might make to comprehend the new spirit of the times, and to give practical effect to modern medical aspirations. Men are beginning to talk of "new wine and old wine-skins" in this connection. The Association, they are saying, has become very large, and like other large creatures it is too unwieldy to move at any but a very slow pace. As a consequence new organisations are springing up by the half-dozen. This may be inevitable, but it is not desirable. The medical profession will speak most effectively when it speaks with one voice. That voice must be living and modern, not a cavernous rattle from a dying throat. This one thing is certain, that there is new wine in modern medicine, in large quantities, and that if the old wine skins have lost all their old elasticity it will burst them. Our hope is that the new wine may find the old skins fresh and elastic, and that so the profession and the state may have the full advantage of a medicine which is both ancient and modern, and therefore both wise and bold.

